# Exploring the religious and spiritual coping experience of cases via cancer: A qualitative research

**Published:** 2015

**Authors:** R Esmaeili, A Hesamzadeh, M Bagheri-Nesami, VL Berger

**Affiliations:** * Orthopedic Research Center, Nasibeh Faculty of Nursing & Midwifery, Mazandaran University of Medical Sciences, Sari, Iran,; ** Nasibeh Faculty of Nursing & Midwifery, Mazandaran University of Medical Sciences, Sari, Iran,; *** Acute Inpatient Rehabilitation Hospital, Houston, Texas, USA

**Keywords:** spiritual coping, oncology care, cancer patients, religion

## Abstract

**Objective:** Lately, the spiritual and religious (S/R) aspect of health behave has been receiving an increased attention in the scientific literature. The study aims to show the components of R/ S coping in cases of cancer in Iran.

**Method: ** The design of the current research is according to a qualitative research using content analysis. Sixteen patients with various kinds of cancer take part in the research. Data was produced though in-depth discussions and the gratified investigation utilized to discover categories and sub-themes.

**Results:** Three categories combined from the information investigation: 1) Maintaining/ improving self-esteem, 2) Positive appraisal/ Being optimistic, and 3) Self-sustaining. In the participants’ view, the general category realized to be the “improving calmness”.

**Conclusions:** Positive R/S coping strategies were used by the patients and most cancer patients tried to achieve calmness through R/ S coping. The findings of the research can be used to plan medical and nursing approach towards increasing the R/S quality care both in the acute and the long-term settings.

## Introduction

There was a growing eager towards the character and influence of mentally and religion in the setting of wellness, disease, and healthcare use [**1**]. There is a mounting evidence that R/S aspects of life are linked via health and wellness. Researchers have attempted to understand the impact of protective resources such as R/S practices and beliefs on health behaviors [**2**]. Spirituality is considered a great harmony experience in that the organism functions by the highest attainable result [**3**]. This describes a holistic personal property which is essential for the person health and wellbeing [**4**]. The term “spiritual” has various applications and purposes, and often the R/ S sizes are conflated, causing the danger of external or visionary strategies to incorporeality [**3**]. Spirituality represents one’s ability to seek purpose and meaning, to make the connection, and to pursue a transcendental value [**5**]. Religion is an expression of spirituality and can be considered as the set of values, beliefs, and practices that people adapt to meet spiritual needs [**6**]. Terms such as religiosity, religiousness, and spirituality are often used interchangeably [**7**]. The R/ S issues are obviously important to adolescents, it is estimated that 95% of the youths suppose in God while 85–95% of them declare that belief is necessary for their life [**8**]. 

Researches investigating the religious dealing in medically sick cases were realized that among 34 and 86 percent of the patients stated application their R/ S cognition and performances in dealing via their disease [**1**]. In addition, between 50% and 95% of the cancer patients consider religion and spirituality personally important and have experienced spiritual needs [**9**]. One team, for whom mentally is a main view requiring a deeper realization consists of the cancer patients [**10**]. Most cancer patients receiving a diagnosis of cancer had an appalling experience [**11**]. In a qualitative study of cancer cases, it was found that majority of the cases had an experience of personal disturbance, describing a feeling of hopelessness and unclear picture of the illnesses in future [**12**]. In fact, viewed via the ambiguity of the current and the numerous uncertainties concerning the next coming, many cancer cases rely on their religious opinions as a reservoir of power [**3**]. Psychological distress occurs frequently at the terminal of living. Therefore, maintenance or developing a discernment of religious wellbeing might be considered a crucial aspect of coping via terminal illness [**13**]. Spirituality is associated with human strength to improve coping with pain, stress, and cancer [**14**].

 Believing coping expressed as ‘‘the application of behavioral and cognitive methods, in the cope with painful living experiences, that arise one’s mentally out’’ [**1**].

Although physicians and nurses have begun to consider a person’s spirituality a notable holistic care view, they usually fail to recognize this view of their patients’ needs [**10**]. The purpose of religiosity as a process of adjustment and a means of coping is important; it is an important component of responsibility for wellness concern suppliers in modern medical rehabilitation [**15**]. Despite a demand for spiritual care that is present in every health care setting, rehabilitation cases usually had clear moral care requires linked to their status [**16**]. The studies on spirituality in the nursing literature are obtained from empiricists who focus on perceptions and practices of patients concerning spiritual needs or care [**4**]. Quantitative studies involving cancer patients and investigating religiousness have yielded mixed results [**17**]. Thune-Boyle and col. (2006), mentioned that the value of spiritual coping via cancer, particularly across various society remains not obvious [**1**]. In a qualitative study by Taleghani and colleagues (2006), on coping techniques in Iranian cancer patients, it realized that the patients used a religious approach to cope via cancer and the authors concluded that religious faith plays a major role facing a diagnosis of cancer [**18**]. This finding was congruent with the research performed by Howard and col. (2007), who showed in their qualitative meta-synthesis research that cancer patients cope with cancer through spirituality [**19**]. The majority of cancer patients receiving palliative care consider themselves spiritual and religious [**20**]. These findings propose the requirement for a more detailed investigation into the dimensions of spiritual deal with in cancer patients. Further study is required to specify the best methods of identifying moral requirements and supplying support to cases in various settings and at various levels of the sickness [**21**]. In their international survey, Selman and col. (2014) indicated that there is a worldwide tendency for research in the domain of spiritual care [**22**]. 

The present research further explains the current debate on the placement of the cancer patients’ spiritual care through the exploration of spiritual coping constructing concepts from the views of these patients in the Iranian context.

## Materials and Methods 

**Study Design**

This qualitative study involves the content analysis method for the ability to offer a systematic coding and categorize an approach by which exploring a large amount of textual information is possible and the reader is provided with a particular view [**23**]. From this position, the researcher is able to make valid inferences from the data in their context, with the purpose of providing knowledge and novel insights [**24**]. Graneheim and Lundman’s (2004) method utilized for analyzing data as it provided a sharp penetration regarding the ideas linked to qualitative investigation and proposed measures throughout the steps of the study [**25**].

Sample

The instance includes of 16 patients (11 females and 5 males) who voluntarily took part in the research and had a diagnosis of cancer. The cases are between 27 and 77 years old (**Table 1**), all patients supplied via purposive sampling of one subspecialty hospital, one subspecialty cancer clinic and one cancer patients aid center, affiliated to Medical Sciences Mazandaran University, Sari, Iran, during the time interval of May to October 2013.

The composition standards for the cases were: (a) diagnosed with cancer, (b) older than 20 years, (c) able to reply the surveys and express their experiment and (d) willing to participate in the study. Over the first contact, partners notified of the purpose and nature of the research and the potential dangers and advantages, and after having their oral approval for assistance, the place and time of the discussion are provided. The recruited patients are confident that they can withdraw at various times and confidentiality was preserved. 

**Table 1:** Demographic properties of the research cases

Participants number	Gender	Age (year)	Type of cancer	Time since diagnosis (Month)	Marital status a	Education b
1	F	35	Breast	6	Sp	BS
2	M	67	Stomach	8	M	Diploma
3	F	39	Breast	12	M	Diploma
4	M	51	Colon	9	M	Diploma
5	M	32	Lymphoma	8	M	AA
6	F	58	Leukemia	4	M	AA
7	F	77	Lymphoma	7	W	Diploma
8	F	27	Thyroid	11	M	Diploma
9	F	75	Colon	8	W	Elementary
10	F	52	Colon	10	M	Diploma
11	F	40	Liver	9	M	Diploma
12	F	42	Breast	2	M	AA
13	M	33	Testis	5	M	Diploma
14	F	50	Breast	8	M	Elementary
15	M	60	Lymphoma	13	M	BS
16	F	53	Colon	1	M	MS
a Marital status: M = married, Sp = separated, W = widow						
b Education: MS = Master of Science, BS = Bachelor of Science, AD = Associate Degree.						

Data collection

After obtaining the ethical approval of Medical Sciences Mazandaran University, the researchers obtained entrance to the possible shareholders. At the commencement of each discussion, simple questions wanted to obtain the participants’ demographic information and to prepare the condition for the below survey. An interview guide was developed by the researchers, which included the following questions: Do you want to tell me how you manage your illness, what things have helped you deal with your illness? Follow-up questions were generated to show the patients’ experience regarding the R/ S aspects of coping during the in-depth interviews. Majority of surveys needs 40-80 minutes and were recorded for a later transcription. Patient recruitment was continued until data saturation.

Analysis of Data 

The recorded voices reproduced exactly. Information investigation initiated after the information got from the original interviews. Each reproduced meeting was held and reread several times to obtain a sense of the entire interview and analyzed line-by-line, entire word to determine code units. Similar codes were linked to the related sub-theme and each sub-theme was allocated to the related themes to manifest the content of the text. The general theme was developed to link the underlying meanings in the themes [**25**].

Trustworthiness

The criteria of Guba and Lincoln (1985) utilized in the current research to ensure trustworthiness. Reliability was founded toward variations in the participants’ age, type of cancer, occupation, gender, and education, in order to a significant order to provide a large representation of the events. The researchers have a continued commitment to the research areas and the writing field notes contributed to data quality. Peer checking performed by two experts who verified the categorization and coding process. There were members who checked nine interview drafts. These were returned to the participants to confirm that the researchers were presenting their actual perceptions. 

Moral concerns

The research confirmed via the Research Ethics Committee of Mazandaran Medical Sciences University in Sari, Iran. Approval was received prior to the enrollment in the research areas. All participants were notified regarding the aims and study nature the as well as the possibility to eliminate of the research at any step, without being penalized. Consent for take part in the research and audiotape recordings were obtained. All the cases were ensured that their confidentiality will be preserved in the research. 

## Results

The investigation of information of all sixteen interviews evidenced results in the three themes of R/ S coping: (1) Maintaining/ improving self-esteem (2) Positive appraisal/ being optimistic (3) Self-sustaining (**Table 2**). These 3 categories were described individually with evidence of instances from the information. All instances specified by numbers, which were assigned in a chronological manner.

**Table 2:** Results of interviews content analysis including general theme, themes, sub-themes

**Main theme**	**Theme**	**Subtheme**
Improving calmness	Maintaining/improving self-esteem	Reinforcing communication with self
		Reinforcing link via god
	Positive appraisal/being optimistic	Giving hope to self
		Positively reframing of the disease
	Self-sustaining	Performing religious rituals
		Avoiding intrusive thoughts

Theme 1: Maintaining/ improving self-esteem

A cancer patient’s R/ S coping strategy through maintaining or improving self-esteem linked to the cases attempt to strengthen communication both with self and God. 

Reinforcing communication with self. 

Most of the cases speak of their striving to talk to themselves to gain assurance. 

“I told myself never mind, if God doesn’t want, anything will happen. They (doctors) tell someone that you’ll live a hundred years, but he won’t be living one day and they tell to another one that you’ll live one day, but he will be alive a hundred years”. (Participant #6, female, 30 years old).

“I told myself that all human beings die one day, any person might get sick and I got sick too” (Participant 12, female, 75 years old).

Reinforcing communication with God

All the participants referred to communication with God. Several declared that they talked directly to Him and sought help from God.

“I really talk to God very much. When I am alone, I talk to God in my loneliness, which gives me a lot of comfort. Now, I have more communication with God, very much”. (Participant #5, female, 42 years old).

“Telling, ‘my God, my God’, became more frequent compared with the past. I talked to my God. Now I have more attention towards God, maybe three times or maybe ten times more” (Participant #3, female, 52 years old). 

“I am only paying attention to God. I am asking those who do not believe in God, what do they do on this occasion and how can they tolerate this situation?” (Participant #16, female, 53 years old).

Theme 2: Positive appraisal/ being optimistic

A large number of participants added that they tried to cope with their disease by means of positive appraisal and being optimistic. They strived to give hope to themselves and interpret the disease positively.

Giving hope to self.

Some of the cases have a dream of remedy and most of them gave hope to themselves. 

“That night I said, show me yourself Imam Reza (a holy person in Islamic history). The same night I dreamt Imam Reza who called me ‘go and do your surgery”. (Participant #3, female, 52 years old).

“I make my effort as far as I can, on my feet, the rest remains to God, whom He knows”. (Participant #10, female, 77 years old).

Positively reframing the disease

A few small numbers of cases have a direct effect to their illness interpretation.

“I say that maybe God has closed a door against me, but he will open hundreds of doors for me”. (Participant #7, female, 27 years old).

“I said that God offered this disease to me to go to a doctor and have a surgery”. (Participant #15, female, 50 years old).

Theme 3: Self-sustaining

In the opinion of the most of the cases, performing religious rituals and avoiding intrusive thoughts are two instruments to deal via the cancer disease.

Performing religious rituals

All cases notified that performing religious rituals such as praying, going to the religious ceremony, visiting shrines, reading the Quran and religious words, had an alleviating effect for them. Several of these religious rituals were also performed by the patients’ family members. 

“I pray days and nights, everyone prays for me” (Participant #1, female, 58 years old).

“My daughter always prays for me, she said ‘Mum, I pray for you”. (Participant #8, female, 27 years old).

“I went to Qum and Mashhad (two holy cities in Iran with two famous shrines) to visit the shrines. I read a religious lament for one month”. (Participant #2, male, 67 years old).

“You know, praying, reading the Quran and performing Namaz (a kind of Islamic praying) day and night do not get me fed up. I perform my Namaz on time” (Participant #4, female, 40 years old).

Avoiding intrusive thoughts

Several participants expressed that the religious belief helped them evade irritating feelings and thoughts.

“I left myself with God; everything that happens would be my fate, everything that God wishes” (Participant #10, female, 77 years old).

“Reading the Quran calms me and I felt that God helped me more. This makes me feel well”. (Participant #8, female, 27 years old).

“I understood that doctors are only tools. Everything is in the hand of God. I left everything to God”. (Participant #9, male, 30 years old).

Improving calmness

Enhancing calmness is the general theme as emerged from data analysis. All the participants used their own way of spiritual coping strategies to reach a satisfactory level of calmness. Trying to reach calmness has been mentioned by the participants directly or indirectly.

“I enjoy communicating with God; it is very effective; it had a direct influence on the disease”. (Participant #9, male, 30 years old).

“I have always wanted God to make me better, to make me well. Reading the Quran helped me forget what I have (cancer)”. (Participant #8, female, 27 years old). 

“I told God, You gave pain and you provided a cure, and then do not delay my cure. In spite of this, I am pleased with his content”. (Participant #12, female, 75 years old).

## Discussion 

In the current research, the cases expressed their experience of R/ S coping strategies. The process analysis and their narratives interpretation revealed that cancer patients believed that they were able to reach calmness through their religious aspect of spiritual coping that included: Maintaining/ improving self-esteem, Positive appraisal/ Being optimistic and Self-sustaining. It is obvious that coping with cancer was a multi-dimensional procedure and the findings of the present study showed that spiritual coping was also multi-dimensional and involved values, belief, and a different of activities. 

The findings revealed that the most of the cases tried to deal with their disease through maintaining/ improving self-esteem. Strengthening communication with self and with God were two major strategies that assisted them to cope with their disease.

Self-esteem is an essential individual source, power linked via the mental functioning [**26**]. Evidence suggests that self-esteem is a primary indicator of health and illness coping [**27**]. In cancer cases, self-esteem might buffer the pressure they test.

But, since the majority researches on cancer cases examined self-esteem as an result parameter, few is identified regarding the self-esteem role in cases mental adjustment [**26**] and coping. Self-esteem plays a central role in the ability of breast cancer survivors to thrive and continue to live “normal” lives [**27**, **28**] and this discussion can be done inside the person’s mind and with God. In a qualitative study conducted by Rahnama (2012), spirituality from the cancer patients’ point of view was defined as the relationship with God, including “mentioning God”, as well as an personal connection via God and the self [**29**]. In their qualitative research on breast cancer patients, Lynn Gall and Cornblat (2002) found that the most of participants with breast cancer described a link via a greater entity, normally specified as God, that had an notable and vital effect in their regulation to cancer and most of these patients had actively changed to and placed on God for provision and help [**30**]. Aflakseir and Coleman (2011) also believe that religious teachings in an Islamic context encourage people to trust and turn to God in times of requirement and for guidance [**31**]. 

Surbone and Baider (2010) believe that reflecting on mentally is identical to shows on “self-identity”. In the procedure of self-development, a done person or persons still in seek for their identity might totally order comparable religious issues and pull power of religious references, usual kept hidden and private from others. Within holiness, we compare via object placed both within and behind us [**3**]. In their qualitative research, Thomas and Retsas (1999) showed that cancer patients tried to “create meaning” and “discover self” in their experience giving them a sense of confidence and empowerment [**10**]. In contrast with this positive view towards a link via God and self, the “spiritual struggle” expressed as “the conflict expressions, survey, and doubt about faith matters, God, and moral and religious links” which are of 3 types: intrapersonal, divine, and interpersonal. Intrapersonal moral struggles were identified by issues and uncertainties regarding mental opinion. Ultimately, divine moral struggles included tensions in the personals link via the divine (or God). Moreover, moral struggles tended to be fewer usual than the direct religious dealing [**32**]. 

According the results of this research, positive appraisal/ being optimistic were recognized as spiritual coping strategies. In confirmation via the current research, in the study of Kurtz et al. (1995), cancer patients who has a direct philosophical/ moral outlook are similar to have good wellness tasks [**33**]. This condition can be the key to long-term cancer adjustment [**34**]. According Gall’s (2000) research results, religious coping behavior was found to correlate with various cognitive appraisals of the current cancer situation, and the God facets of benevolence, challenge, and presence were positively related to perceiving the illness as having some gain and importance/ meaning to life. Optimistic dealing methods included the application of positive thoughts, keeping a positive outlook, and made positive comparisons [**35**]. Optimism acted as a source that maintained a positive condition, protecting personals from the possible adverse impacts of cancer and its therapy [**36**]. Lauver and Tak (1995) found that optimism linked via a fewer delay and pressure in care searching and via desirable outcomes expectations of care searching in cancer cases [**37**]. Studies of optimism in cancer cases have shown that it was directly linked to mental well-being [**38**,**39**]. Hope was expressed as a vital part in living. Having things to hope for was a vital dealing method for terminally sick cancer cases [**40**].

Aquino and Zago (2007) asserted that their study participants had the “hope for the second opportunity” and the religious beliefs fulfilled the requirement for hope in the upcoming events [**41**].

 Rahnama et al. claimed that their study participants mentioned that religious opinions about the feasibility of advancement by God’s will and miracles were also amongst the religious sources – fueling their hope of survival [**29**]. Irving and colleagues (1998) found in their study that hope is a means of maintaining a “fighting spirit” for dealing with it [**42**, **43**].

 According to Lutgendorf et al. (2002), cancer patients who coped with their disease using positive reframing reported better functional, emotional, and physical well-being, and higher overall quality of life [**44**]. Thornton and Perez (2006) also reported that for cancer survivors, coping by using positive reframing was linked via higher stages of posttraumatic growth. Concomitantly with the passage of time since cancer treatment initiation, several patients learnt to reframe the difficulty of survivorship to a chance for individual increase [**45**].

Based on the findings of this research, nearly all the participants used self-sustaining strategies to deal via their disease. According to a self-sustaining model that was initially introduced for young adults, adolescent patients regaled to the issues of the cancer skill via starting certain behavioral and cognitive dealing methods (distraction). The self-sustaining procedure described as a general progression through which adolescents who are testing real wellness threats move to comfort themselves and to reach competence in solving wellness dangers [**46**]. Although this phenomenon was first introduced for youngsters with cancer, it appears that more study is required to elucidate the function of this element in adults who suffer from cancer. 

Similarly to the current study, in a research performed by Rahnama (2012), the participants described that they have done religious activities involving “saying prayers (Namaz)”, “visiting the shrines and holy area”, “mentioning God” [**29**]. Guz and colleagues (2012), mentioned in their research findings that cancer patients engaged in several religious and spiritual activities such as praying and visiting a tomb [**47**]. Aquino and Zago (2007), revealed in their research that the cancer patients emphasized the religious behavior such as collective or individual pray, praying the rosary, going to church, talking to the minister [**41**].

The intrusive thoughts experience has been linked to greater emotional anxiety and reduced state of living in cancer cases both during treatment and during post-treatment [**48**, **49**]. Helgeson and Lepore (1998) indicated that there is a powerful negative relation between intrusive thoughts and emotional health among prostate cancer patients who felt socially constrained in speaking regarding it compared to patients who felt unconstrained [**50**]. 

We found that improving calmness is the important category that emerged of our data analysis.

Compatible with the current study, the research result of Lundberg and Trichorb (2001) indicated that the most common feelings of cancer patients of both genders at first knowledge about their treatment were “acceptance and calmness” [**51**]. According to Lynn Gall and Cornblat (2002), the R/ S opinion in a greater power displaye to be a relatively stable source for most cancer patients that were intricately intertwined in the fabric of the way they found cancer. The link via God acted a functions variety for these cases, involving increasing calmness [**30**]. In their review article, Visser et al. (2010) mentioned that typical R/ S trainers like meditation and prayer were realized to be linked via reduced pressure of blood, advanced functioning of immune, advanced heart rate variability, and vital in this context, a general sense of relaxation and calmness [**52**]. 

Patients with cancer in rehabilitation settings require hope and support to help them improve their well-being. The R/ S issues seem to be very significant in the procedure of dealing for many cancer patients. The participants’ experience in the current research demonstrated that all of them put stress on the direct view of R/ S coping strategies that helped them cope with their disease. The results of the current research appeared to be remarkable, as they described positional R/ S components of dealing via cancer in patients. Furthermore, health practitioners, especially rehabilitation nurses could assist their cancer patient’s deal via their life-threatening illness through delivering the needed conditions and providing suitable environments for using those R/ S resources to cope via their problem.

Although this research was done on Muslim Iranian patients with no concentrate on a particular kind of cancer, the results had the potential to be considered a resource of promise in both practice and research fields and it is suggested to do alike researches on a particular kind of this problem and in other religions and contexts.

**Acknowledgments**

The authors acknowledge the assistance of the Deputy of Research and Technology of Mazandaran University of Medical Sciences for funding the research (grant number: 92-14). The genuine participation of the cases in the study is also appreciated. 
